# Flame-Made Nb-Doped TiO_2_ Ethanol and Acetone Sensors

**DOI:** 10.3390/s110100472

**Published:** 2011-01-05

**Authors:** Sukon Phanichphant, Chaikarn Liewhiran, Khatcharin Wetchakun, Anurat Wisitsoraat, Adisorn Tuantranont

**Affiliations:** 1 Materials Science Research Center, Faculty of Science, Chiang Mai University, Chiang Mai, 50200, Thailand; 2 Department of Physics and Materials Science, Faculty of Science, Chiang Mai University, Chiang Mai, 50200, Thailand; E-Mail: chaikarn_l@yahoo.com (C.L.); 3 Nanoscience and Nanotechnology Program, Graduate School, Chiang Mai University, Chiang Mai, 50200, Thailand; E-Mail: to_khatcharin@hotmail.com (K.W.); 4 National Electronics and Computer Technology Center, Pathumthani, 12120, Thailand; E-Mails: anurat.wisitsoraat@nectec.or.th (A.W.); adisorn.tuantranont@nectec.or.th (A.T.)

**Keywords:** TiO_2_, niobium, flame spray pyrolysis, acetone and ethanol sensor

## Abstract

Undoped TiO_2_ and TiO_2_ nanoparticles doped with 1–5 at.% Nb were successfully produced in a single step by flame spray pyrolysis (FSP). The phase and crystallite size were analyzed by XRD. The BET surface area (*SSA*_BET_) of the nanoparticles was measured by nitrogen adsorption. The trend of *SSA*_BET_ on the doping samples increased and the BET equivalent particle diameter (*d*_BET_) (rutile) increased with the higher Nb-doping concentrations while *d*_BET_ (anatase) remained the same. The morphology and accurate size of the primary particles were further investigated by high-resolution transmission electron microscopy (HRTEM). The crystallite sizes of undoped and Nb-doped TiO_2_ spherical were in the range of 10–20 nm. The sensing films were prepared by spin coating technique. The mixing sample was spin-coated onto the Al_2_O_3_ substrates interdigitated with Au electrodes. The gas sensing of acetone (25–400 ppm) was studied at operating temperatures ranging from 300–400 °C in dry air, while the gas sensing of ethanol (50–1,000 ppm) was studied at operating temperatures ranging from 250–400 °C in dry air.

## 1. Introduction

TiO_2_ is used extensively as a gas sensing material due to its change in electrical conductivity under analyte gas exposure. Sensing capability has been improved with the addition of foreign atoms such as Cr [1], Mo and W [2], Pt and Nb [3], Fe [4], and La and Cu [5]. Nb doping of TiO_2_ has been used for O_2_, CO, NO_2_, and ethanol sensing. Nb doping modifies the microstructure of TiO_2_, controls grain growth mechanisms, introduces electronic defects at the surface or in the bulk of grains and so modifies TiO_2_ conductivity and gas sensing. TiO_2_ presents three crystalline structures: brookite, anatase, and rutile.

The Nb_2_O_5_-TiO_2_ system has been prepared by several methods such as the solid state reaction of Nb_2_O_5_ and TiO_2_ [6–8], sol-gel [9,10], RF-sputtering of thin films [11–13], laser induced pyrolysis [14], pulsed laser deposition [15] and thick film using powder screen printing [16]. The Nb_2_O_5_-TiO_2_ system can be used in applications such as varistors [17], catalysts [18], photocatalysts [19,20], and electrodes applicable to photoelectonic devices such as *p-n* type solar cells [21] and hybrid solar cells [22]. For gas sensing applications, it has been reported that the Nb_2_O_5_-TiO_2_ system shows higher sensitivity and shorter response time as an oxygen gas sensor than undoped TiO_2_ [11]. The Nb_2_O_5_-TiO_2_ system can be used for sensing other gases as well, such as CO [23], CO, and ethanol [9,16,24], CO and NO_2_ [16,24], and ethanol [3]. Table 1 lists literature examples of the use of Nb-TiO_2_ for gas sensing applications, showing the authors, method of preparation, % Nb, sensing gas, range of detection, type of titania, size, and some remarks.

Comini *et al*. [3] reported that Nb- and Pt-doped TiO_2_ thin films could be used for ethanol and methanol sensors. The thin films were prepared using the sol-gel process by the spin coating technique on Al_2_O_3_ substrate. The sensors were tested under exposure of ethanol and methanol gases at 300 °C with the concentration ranging from 500–1,250 ppm, making them feasible for development of breath analyzers (detection limit is 200 ppm). The thicknesses of the film were ranging from 60–100 nm. It was noticed that 1 at.% Nb and 0.5 at.% Pt/TiO_2_ showed the best sensing performance. The TiO_2_ sensors developed were sensitive at up to 500 ppm of ethanol. The response and recovery dynamics to ethanol were particularly promising for applications in food analysis, electronic noses, and breath analyzers.

In comparison to the same materials, Teleki *et al*. [16] reported the preparation of a flame-made TiO_2_ spherical particles film of about 30 μm thickness by drop-coating of a heptanol suspension of these powders, and sensing tests at 500 °C with ethanol at concentrations ranging from 10–75 ppm. The sensor showed the highest sensor signal at 75 ppm (*S* = 30) ethanol concentration. Secondly, Teleki *et al*. [23] reported in 2008 the effect on ethanol and CO gas sensing of flame-made Nb- and Cu-doped TiO_2_ thick film (5 μm) sensors fabricated by doctor-blading. All sensors were tested with gas concentrations ranging from 25–300 ppm during forward and backward cycles at 400 °C. Niobium stabilized the anatase phase and retarded grain growth up to 600 °C. The sensitivity increased by addition of either Cu and Nb to titania, and the best improvement was found for the 4 at.% Nb/TiO_2_ sensor. Decreasing the Nb concentrations from 10 to 4 at.% increased the response. The response of 10 at.% Nb/TiO_2_ sensor was high, but the baseline was not stable. The response time decreased with increased ethanol concentration, from 180 to 15 s for 25 and 300 ppm, respectively. The recovery time was very slow, within the 5–10 min range. This, however, decreased with increasing ethanol concentration. The anatase phase seems to be crucial as the Nb/TiO_2_ sensor showed the highest response while Cu doping had no influence on the response relative to undoped TiO_2_.

Flame spray pyrolysis (FSP) is a very promising technique for synthesis of high purity nanosized materials with controlled size and high surface area in one step. FSP has been demonstrated to produce high surface area of tin dioxide nanoparticles for gas sensing [24]. The aim of this research was to apply this technique to synthesize niobium-doped TiO_2_ nanoparticles. Characterization of the nanoparticles and their acetone and ethanol sensing properties were performed.

## 2. Experimental

### 2.1. Flame Synthesis of Nanopowders

**Scheme 1.**
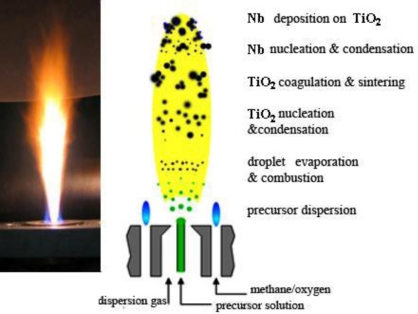
The formation of Nb-doped TiO_2_ by flame spray pyrolysis.

Titanium isopropoxide (Aldrich, 97%) and niobium (IV) 2-ethlyhexanoate (Strem Chemicals) were used as titanium and niobium precursors, respectively. Both precursors were dissolved in xylene (Fluka, >98.5%) and acetonitrile (Fluka, >99.5%) in equal volume with the total metal atom concentration maintained at 0.5 mol/L. The niobium concentration was varied between 1 and 5 at.%. The precursor was fed into a flame spray pyrolysis reactor [24] by a syringe pump (Inotech) with a rate of 5 mL/min and was dispersed into droplets by 5 L/min of oxygen (Pan Gas, purity > 99%) using a gas assisted nozzle. The pressure drop at the nozzle tip was kept at 1.5 bar. The water-cooled system of the reactor avoided any evaporation of the precursor within the liquid feed lines or overheating of the nozzle. The spray flame was maintained by a concentric supporting flamelet ring of premixed methane/oxygen (CH_4_ 1.5 L/min, O_2_ 3.2 L/min). In order to assure the presence of enough oxidant for complete conversion of the reactants, an additional outer oxygen flow (5 L/min) was supplied. The powder was collected with the aid of a vacuum pump (Vaccubrand) on a glass fiber filter (GF/D Whatman, 25.7 cm in diameter). During the experiment, the filter was placed in a water-cooled holder, 40 cm above the nozzle, keeping the off-gas temperature below 200 °C. Scheme 1 shows the formation of Nb-doped TiO_2_ by flame spray pyrolysis.

### 2.2. Powder Characterization

X-ray diffraction (XRD) patterns were recorded with a Bruker AXS D8 Advance (40 kV, 40 mA) operating with Cu K_α_. The relative amounts of anatase and rutile and their respective crystallite sizes were calculated from the XRD data using the Rietveld method. BET powder-specific surface area (*SSA*), was measured by nitrogen adsorption at 77 K (Micromeritics Tristar) after degassing the sample for 1 h at 150 °C in nitrogen. The equivalent average primary particle diameter *d*_BET_ was calculated by *d*_BET_ = 6/(*SSA* ρ_P_). Here, ρ_P_ is the average density of TiO_2_ calculated from weight percent and density of anatase and rutile where *d*_anatase_ and *d*_rutile_ are 3.97 g/cm^3^ and 4.17 g/cm^3^ respectively. Morphologies of all the flame-made powders were investigated by Transmission Electron Microscopy (TEM, Hitachi H600, operated at 100 kV).

### 2.3. Paste and Sensor Preparations

An appropriate quantity of homogeneous mixed solution (0.28 mL) was prepared by stirring and heating at 80 °C for 12 h ethyl cellulose (Fluka, 30–70 mPa·s) as the temporary binder and terpineol (Aldrich, 90%) as a solvent. The liquid mixture was combined with 60 mg of samples 1–5 at.% Nb/TiO_2_ nanopowders and mixed for 30 min to form a paste prior to spin-coating. The resulting paste was firstly spin-coated (700 rpm) 1 time for 10 s, and then subsequently at 3,000 ppm, 2 times for 30 s on the Al_2_O_3_ substrates interdigitated with Au electrodes (0.5 × 0.5 cm) to deposit sensing films. The resulting substrates were annealed in an oven at 150 °C for 1 h with an annealing rate of 1 °C/min and at 400 °C for 1 h with an annealing rate of 1 °C/min for binder removal prior to the sensing test.

### 2.4. Sensor Measurement

The sensor characteristics of the sensing films were determined with acetone (25–400 ppm) and ethanol (50–1,000 ppm). The flow through technique was used to test the gas-sensing properties of sensing films. A constant flux of synthetic air of 2 L/min as gas carrier was flowed to mix with the desired concentration of pollutants dispersed in synthetic air. All measurements were conducted in a temperature-stabilized sealed chamber at 20 °C under controlled humidity. The gas flow rates were precisely manipulated using a computer controlled multi-channel mass flow controller. The external NiCr heater was heated by a regulated DC power supply to different operating temperatures. The operating temperature was varied from 250 °C to 400 °C. The resistances of various sensors were continuously monitored with a computer-controlled system by voltage-amperometric technique with 5 V DC bias and current measurement through a picoammeter. The sensor was exposed to the gas mixed sample for ∼5 min for each gas concentration testing and then the air flux was restored for 15 min. The response (*S*) is defined in the following as the resistance ratio *R*_a_/*R*_g_ [25], where *R*_a_ is the resistance in dry air, and *R*_g_ is the resistance in the test gas. The response time (*T*_res_) is defined as the time required until 90% of the response signal is reached. The recovery time (*T*_rec_) denotes the time needed until 90% of the original baseline signal is recovered [25]. After the sensors fabricated using samples undoped TiO_2_, 1 at.% Nb/TiO_2_, 3 at.% Nb/TiO_2_, and 5 at.% Nb/TiO_2_ had been tested with varied operating temperatures, they were designated as S0, S1, S3, and S5, respectively.

## 3. Results and Discussion

### 3.1. Nanopowder Properties

**Figure 1.**
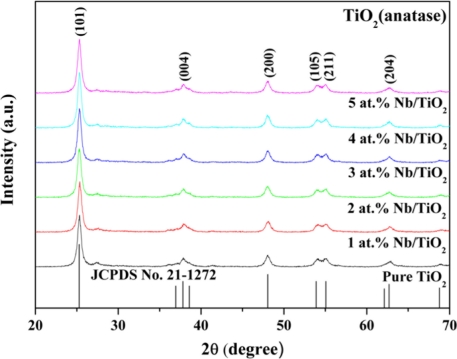
XRD patterns of undoped and Nb-doped TiO_2_ flame-made samples.

The XRD technique was used to study the relative amounts of anatase and rutile phases and the evolution of crystallite sizes as a function of composition of the flame-made undoped TiO_2_ and Nb-doped TiO_2_. Figure 1 shows the XRD patterns of nano-sized undoped TiO_2_ and 1–5 at.% Nb/TiO_2_ samples. The nanopowders were highly crystalline, and all peaks can be confirmed to be the anatase phase (JCPDS file no. 21-1272). No amorphous phase and the characteristic peaks attributed to Nb or NbO_2_ were found in the XRD patterns. It can be assumed that the amount of Nb doping particles was very low, which resulted in non-appearance of the Nb peaks. The relative amounts of anatase and rutile and their respective crystallite sizes were calculated from the XRD data using the fundamental parameter approach Rietveld method [26]. The average crystal sizes (*d*_XRD_ ave.) were calculated based on the half-maximum widths in Scherrer equation [27] using the TOPAS-3 software, which compared with the average BET-equivalent particle diameter (*d*_BET_) as shown in Table 2. The *d*_XRD_ (rutile) was slightly increased with increasing Nb-doped concentrations. On the other hand, the *d*_XRD_ (anatase) decreased with increasing Nb-doping concentrations. The rutile weight fraction percentage and *d*_BET_ remained almost the same with increasing Nb concentrations. It can be concluded from Table 2 that (1) *d*_XRD_ anatase were smaller than *d*_XRD_ rutile (2) *d*_BET_ and *d*_XRD_ anatase were not affected by the amount of dopant but *d*_XRD_ rutile were.

**Figure 2.**
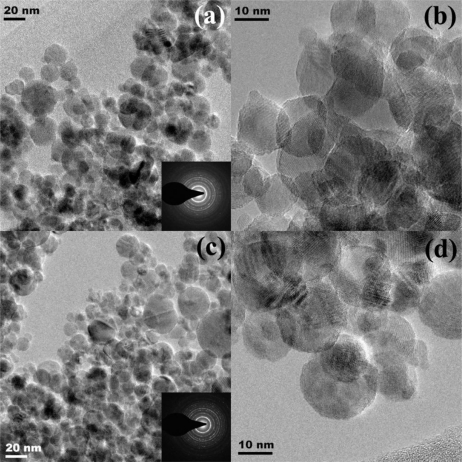
**(a,b)** HR-TEM bright-fields images of highly crystalline flame-made (5/5) TiO_2_ nanoparticles and **(c,d)** 5 at.% Nb/TiO_2_ nanoparticles with different magnifications. Insets show the corresponding diffraction patterns of the particles and clearly the TiO_2_ lattice planes.

Figure 2 shows HR-TEM bright-field images of (a,b) undoped TiO_2_ and (c,d) 5 at.% Nb/TiO_2_ nanoparticles with different magnifications. The corresponding diffraction patterns were shown in the insets. Both samples were highly crystalline as seen from the intense electron diffraction patterns (Figure 2(a,c): insets), which were in good agreement with the XRD data. Figure 2(a–d) show the TEM bright-field images of the FSP-made (5/5) nanoparticles, which were aggregated of primary particles. Nb-doped TiO_2_ nanopowder formed Ti_1−x_Nb_x_O_2_ with a fully solid solution due to diffusion of Nb atom into the TiO_2_ nanoparticles because Nb^4+^ has a similar ionic radius (0.64 Å) to Ti^4+^ (0.605 Å). Teleki *et al*. [23] reported that niobium was partly incorporated in the titania lattice promoting anatase formation.

Figures 2(a,b) show the morphologies of flame-made (5/5) undoped TiO_2_ and 5 at.% Nb/TiO_2_ nanoparticles containing spherical nanoparticles with average diameters of 13 and 11 nm, respectively. The primary particle diameters observed by TEM were consistent with both the *d*_BET_ and the *d*_XRD_. Particularly, the lattice fringes of 5 at.% Nb/TiO_2_ nanoparticles were also clearly visible in a HRTEM image at higher magnification (Figures 2(b,d)). TEM bright-field images can reveal internal structure and a more accurate measurement of particle size and morphology.

### 3.2. Gas Sensing Properties

**Figure 3.**
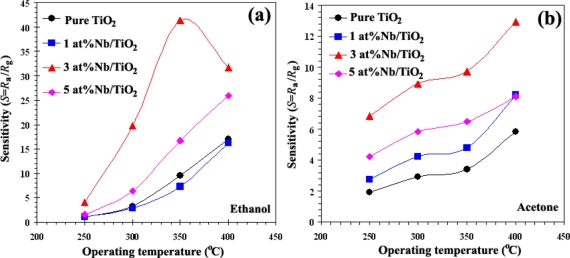
The sensitivity as a function of sensor operating temperature for undoped and doped with different Nb concentrations (1, 3, and 5 at.% Nb) for **(a)** ethanol and **(b)** acetone vapors in dry air atmosphere.

The gas sensitivity is usually dependent on the sensor operating temperature and the dopant. Figure 3 shows the response as a function of sensor operating temperature for undoped and doped with different Nb concentrations (1, 3, and 5 at.% Nb) for ethanol (Figure 3(a)) and acetone (Figure 3(b)) vapors in dry air atmosphere. The measurement of the resistance *vs.* temperature (R/T) profile of undoped TiO_2_ sensor revealed a strong temperature dependence of their resistance and quite low response. It was evident from these results that the response of the undoped sensor (S0) was very poor compared to the doped sensors in different concentration concentrations. With 3 at.% Nb doped TiO_2_ sensor (S3), the sensor showed maximum response to ethanol vapor at an operating temperature of 350 °C for sensor S3 and 400 °C for the other sensors, and for acetone vapor maximum sensitivity at an operating temperature of 400 °C for all sensors. The best sensitivities can be seen at the highest concentration of gases (to 1,000 ppm; *S*_eth_ = 41.4 (350 °C), *S*_eth_ = 31.7 (400 °C), to 400 ppm, *S*_acet_ = 13.0) and response time was extremely fast about 1 s (400 °C) and 9 s (350 °C) for ethanol vapor and of about 33 s for acetone vapor. On the other hand, the response decreased at higher Nb concentration (5 at.% Nb/TiO_2_; S5). Further increase of the dopant concentration decreased the ethanol and acetone sensitivity and deteriorated the response time. The optimum concentration of Nb doping on TiO_2_ sensor was found to be 3 at.% Nb. Possibly a segregated Nb phase was formed on the surface at a higher Nb content (5 at.% Nb/TiO_2_). For low Nb content (3 at.%), Nb nanoparticles are very small compared to TiO_2_ nanoparticles and they can be well dispersed on TiO_2_ nanoparticles. Thus, Nb nanoparticles are very effective catalyst. In contrast, larger Nb nanoparticles, which are formed at higher Nb contents, cannot be well dispersed and cause possible separation among TiO_2_ nanoparticles. Therefore, catalytic action of Nb becomes considerably less effective. This is the reason why the gas sensitivity decreases significantly at the higher Nb content of 5 at.%.

Traversa *et al*. [9] reported a solubility of up to 5 at.% Nb in anatase TiO_2_. They attributed this to small segregated crystalline domains of niobia (Nb_2_O_5_), which were not visible in the TEM bright-field images (Figure 2). Our results agreed well with those of Comini *et al*. [28] showing that Nb doping improved the response to ethanol with respect to undoped TiO_2_. Teleki *et al.* [23] reported that 4 at.% Nb/TiO_2_ showed higher response towards 25–300 ppm ethanol than 10 at.% Nb/TiO_2_ at 400 °C.

**Figure 4.**
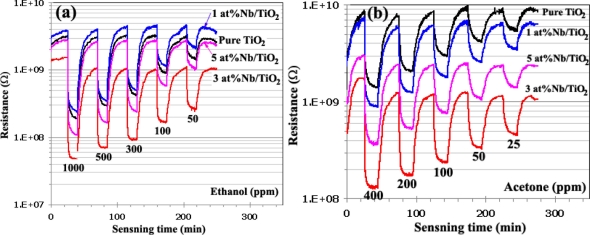
Change in resistance of sensors under exposure to reducing **(a)** ethanol and **(b)** acetone gases during backward cycle with various concentration of ethanol (50–1,000 ppm) and acetone (25–400 ppm) in dry air at 400 °C.

Figures 4(a,b) show the change in resistance of sensor S0 (Undoped TiO_2_), S1(1 at.% Nb/TiO_2_), S3 (3 at.% Nb/TiO_2_), and S5 (5 at.% Nb/TiO_2_) under exposure to reducing gas ethanol (Figure 4(a)) and acetone (Figure 4(b)) at the concentration ranging from 50–1,000 ppm and 25–400 ppm, respectively with the same operating temperature of 400 °C during the backward cycle. The original baseline (dry air) of ethanol sensing was stable during the sensing test. The resistance drastically decreased during the gas exposure with increasing VOC analyte gas concentration, typical for anatase TiO_2_ as an n-type semiconductor. Nb-doped TiO_2_ could exhibit a stronger *n*-type character and a higher electronic conductivity than undoped TiO_2_ as the electron concentration in the titania lattice might increase and the Fermi level might be shifted closer to the conduction band level. The stabilized original baselines of sensors led to sensor response accuracy in terms of sensitivity and response time detection. The base-resistance of the sensor S3 (3 at.% Nb/TiO_2_) was the lowest compared to the other sensors. This is because the sensor S3 had the appropriate amount of concentration and also could perform the sensing properties on the surface and by bulk interaction. The gas sensing behavior of semiconducting oxide sensors could be attributed to both regions of surface and bulk interactions, depending on the small grain size and the appropriate thickness of sensing films. The effects of a high conductivity of the sensor are described clearly from the interactions between VOCs gases and the surface-absorbed oxygen species such as peroxide ion $\left(O_{2}^{2-}\right)$ and superoxide ion $\left(O_{2}^{-}\right)$. These reactions produce more electrons and thus increased the conductivity of TiO_2_ upon exposure to ethanol and acetone vapor.

**Figure 5.**
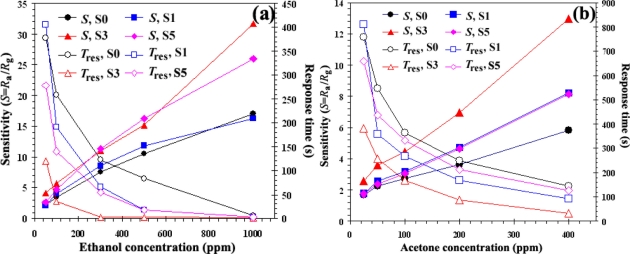
Sensitivity of S0, S1, S3, and S5 (filled symbols, left axis) and the corresponding response time (open symbols, right axis) of **(a)** ethanol and **(b)** acetone detections.

Figures 5(a,b) show the plots of response (*S*) and response times (*T*_res_) *versus* the ethanol and acetone vapor concentrations ranging from 50–1,000 ppm and 25–400 ppm plot for the sensors S0, S1, S3, and S5 during the backward cycle at the operating temperature of 400 °C. Because the particle size of TiO_2_ was in the nanometer range and Nb is known as an excellent catalyst for VOC gases, we paid close attention to the gas sensing activity of this material. From the data, the response of all Nb doping concentrations (S1, S3, and S5) appeared to be higher than that of an undoped TiO_2_ sensor (S0). The response of both gases (filled symbols, left axis) increased linearly and the response time (open symbols, right axis) decreased drastically with increasing ethanol and acetone concentrations. Moreover, it was found that the 3 at.% Nb concentration (S3) sensor showed the best sensing performance in terms of response (*S* = 31.7) and response time. The response time of 3 at.% Nb/TiO_2_ sensor (S3) for 1,000 ppm at 400 °C was very fast—within 1 s (open triangles, right axis)—which was better than that of undoped TiO_2_ (6 s) (open circles, right axis) and other doping concentrations (1 at.% Nb/TiO_2_ = 3 s (open rectangles, right axis), and 5 at.% Nb/TiO_2_ = 2 s (open diamonds, right axis)). The fast response time suggests a surface controlled sensing mechanism, where a steady-state adsorption of ethanol and desorption of CO_2_ [25] on the sensing films was rapidly reached. This is the common interaction between the reducing gas ethanol and surface-adsorbed oxygen species of sensing layer including ethanol with those of its oxidation products (CO_2_ and H_2_O) *versus* times in dry air. This is because CO_2_ was the majority product oxidized with oxygen on the surface of semiconductor materials. This indicates a partial combustion of ethanol to CO_2_ and H_2_O, as well as a release of ethoxides formed during the adsorption of ethanol on the sensing surface. Also, Nb possible increases the number of surface-adsorbed oxygen species, thus promoting reaction sizes for CO oxidations. Teleki *et al*. [23] reported the response of 30 with response time of 375 seconds towards 75 ppm ethanol at 500 °C for undoped TiO_2_ sensing film by the drop-coating technique.

With the acetone response, the sensing performances were lower than for ethanol vapor in terms of the sensor response, sensitivity, and response time. It was noticed that the Nb concentration (3 at.%; S3) sensor showed the best sensing performance at an operating temperature of 400 °C for the highest acetone concentration to 400 ppm in terms of response (*S* = 13.0) and response time. The response time of acetone sensors were with a few minutes. The best response time of 3 at.% Nb/TiO_2_ sensor (S3) for 400 ppm at 33 s which was better than undoped TiO_2_ (147 s) (open circles, right axis) and the other doping concentrations (1 at.% Nb/TiO_2_ = 93 s (open rectangles, right axis), and 5 at.% Nb/TiO_2_ = 126 s (open diamonds, right axis)). The response time of 3 at.% Nb/TiO_2_ sensor (S3) for 400 ppm at 400 °C was slightly sluggish compared to 300 ppm of an ethanol (2 s) (Figure 5(a), open triangles, right axis) sample. Doping the TiO_2_ with 3 at.% Nb resulted in a much steeper calibration curve and the highest sensor signal compared to undoped TiO_2_ (see Figures 5(a,b)). The higher sensor signal and especially the higher response (*i.e.*, the steeper response curve) increased sensor performance.

## 4. Conclusions

FSP was successfully used for the preparation of undoped TiO_2_ and 1–5 at.% Nb/TiO_2_ nanopowders for application to acetone and ethanol gas sensing. The trend of *SSA*_BET_ on the doping samples increased and the *d*_XRD_ (rutile) increased with the higher Nb-doping concentrations while *d*_BET_ remained almost the same. Nb could form a solid solution in the crystal structure of TiO_2_ due to the fact the size of Nb^5+^ (0.64 Å) is similar to that of Ti^+4^ (0.605 Å), thus the size of particles in the doping samples were not affected by Nb atoms as shown from the HRTEM. The crystallite sizes of undoped and Nb-doped TiO_2_ spherical were in the 10–20 nm range. The gas sensing of acetone (25–400 ppm) was studied at operating temperatures ranging from 300–400 °C in dry air while the gas sensing of ethanol (50–1,000 ppm) was studied at operating temperatures ranging from 250–400 °C in dry air. The 3 at.% Nb-dispersed on TiO_2_ sensing film showed a response of 31.7 and a very fast response time of 1 second towards 400 ppm ethanol, as compared to an undoped TiO_2_ sensing film. The 3 at.% Nb-dispersed on TiO_2_ sensing film also showed a response of 13 and a response time of 33 seconds towards 400 ppm acetone. The response times in our study were faster than the previously reported values [16,23]. The highest responses for acetone and ethanol occurred at 400 and 350 °C, respectively.

## Figures and Tables

**Table 1.** Literature review on Nb-TiO_2_ for gas sensing applications showing the authors, method of preparation, % Nb, sensing gas, range of detection, type of titania, size and some remarks.

**Authors**	**Method**	**% Nb**	**Gas**	**Range**	**Titania**	**Size**	**Remarks**
Sharma *et al*. [7]	Thick film using screen printing of powder at 1,300 °C for 5 h	0, 0.2, 0.4 wt.% Nb	O_2_	1,200 ppm Nb-doped 1,000 ppm Cr-doped	Rutile	0.7 mm Nb-doped, 1–5μm Cr-doped	Highest sensitivity of Nb-doped at 550 °C and Cr-doped at 700 °C

Bonini *et al.* [16]	Laser induced Pyrolysis powders Screen printing at 650–1,050 °C	10 at.% Nb, Ta, Ga	CO NO_2_	100 ppm	Anatase + rutile	50–150 nm	Addition of dopants inhibits grain growth and hinders conversion of anatase to rutile

Ruiz *et al.* [8]	Sol-gel powders	0, 2, 4, 6, 8, 10 at.%	CO EtOH	CO 0–1,000 ppm EtOH 0–150 ppm	Rutile 100% (0 at.% Nb) Anatase 87% (10 at.% Nb)	8.5 nm (6 at.%Nb) 10 nm (10 at.%·Nb)	CO sensitivity increases with addition of Nb, EtOH is slightly inhibited

Traversa *et al*. [9]	Sol-gel powders, Screen printing	0, 5, 10 at.% Nb, Ta	CO	0.5 ppm	Anatase (400 °C) Rutile(850 °C)	200–600 nm	Ta and Nb inhibit anatase to rutile transformation

Teleki *et al*. [16]	FSP	undoped	EtOH	1–75 ppm	Anatase + rutile	20–50 nm	Highest sensor signal at 75 ppm (*S* = 30)

Teleki *et al.* [23]	FSP	4, 10 at.%	CO EtOH	25–300 ppm	Anatase + rutile		4 at.% Nb gave higher sensitivity towards EtOH at 400 °C

**Table 2.** The relative amounts of anatase, rutile and their crystallite sizes calculated from the XRD data using the Rietveld method and BET average primary particle size of undoped TiO_2_ and Nb-doped TiO_2_ FSP samples.

**at.% Nb**	**Anatase (wt.%)**	**d_XRD_, Anatase (nm)**	**Rutile (wt.%)**	**d_XRD_, Rutile (nm)**	**d_BET_ (nm)**
0	86.3	17.9	13.7	12.5	15.0
1	86.7	17.5	13.3	10.9	14.1
2	88.1	17.7	11.9	11.3	14.0
3	90.5	18.1	9.5	13.6	14.3
4	90.9	18.8	9.1	14.3	14.8
5	93.4	17.3	6.6	15.2	14.4
